# Acupuncture as analgesia for low back pain, ankle sprain and migraine in emergency departments: Study protocol for a randomized controlled trial

**DOI:** 10.1186/1745-6215-12-241

**Published:** 2011-11-15

**Authors:** Marc Cohen, Shefton Parker, David Taylor, De Villiers Smit, Michael Ben-Meir, Peter Cameron, Charlie Xue

**Affiliations:** 1 RMIT University Australia PO Box 71 Bundoora, Victoria 3083 Australia; 2 Austin Health PO Box 5555, Heidelberg, Victoria 3084 Australia; 3 Alfred Health 55 Commercial Road, Melbourne, Victoria 3000 Australia; 4 Cabrini Health 183 Wattletree Rd, Malvern, Victoria 3144 Australia

**Keywords:** Acupuncture, pain, ankle sprain, migraine, low back pain, emergency, acute

## Abstract

**Background:**

Pain is the most common reason that patients present to an emergency department (ED) and is often inadequately managed. Evidence suggests that acupuncture is effective for pain relief, yet it is rarely practiced in the ED. The current study aims to assess the efficacy of acupuncture for providing effective analgesia to patients presenting with acute low back pain, migraine and ankle sprain at the EDs of four hospitals in Melbourne, Australia.

**Method:**

The study is a multi-site, randomized, assessor-blinded, controlled trial of acupuncture analgesia in patients who present to an ED with low back pain, migraine or ankle sprain. Patients will be block randomized to receive either acupuncture alone, acupuncture as an adjunct to pharmacotherapy or pharmacotherapy alone. Acupuncture will be applied according to Standards for Reporting Interventions in Clinical Trials of Acupuncture (STRICTA). Pain after one hour, measured using a visual analogue scale (VAS), is the primary outcome. Secondary outcomes measures include the following instruments; the Oswestry low back pain disability questionnaire, 24-hour Migraine Quality of Life questionnaire and Patient's Global Assessment of Ankle Injury Scale. These measures will be recorded at baseline, 1 hour after intervention, each hour until discharge and 48 ± 12 hours of ED discharge. Data will also be collected on the safety and acceptability of acupuncture and health resource utilization.

**Discussion:**

The results of this study will determine if acupuncture, alone or as an adjunct to pharmacotherapy provides effective, safe and acceptable pain relief for patients presenting to EDs with acute back pain, migraine or ankle sprain. The results will also identify the impact that acupuncture treatment may have upon health resource utilisation in the ED setting.

**Trial registration:**

Australia and New Zealand Clinical Trials Register (ANZCTR): ACTRN12609000989246

## Background

There is evidence that pain is often inadequately managed in emergency settings [1,2]. Emergency department (ED) staff is constantly under pressure to assess, treat and admit or discharge patients within strict time constraints despite having little or no control over patient influx [1-3]. Hence, additional therapy that could provide safe and effective pain relief, shorten ED length of stay, and avoid patient admission would be advantageous.

Since 1984, acupuncture has been reimbursed by the Australian National health System under the Medicare Benefits Scheme (MBS) (items 173, 193, 195, 197 and 199). In the 2009/2010 financial year, there were a total of 537,785 acupuncture services reimbursed by Medicare at a cost of $ 21,143,968. Of these services, only 49 were delivered within hospitals at a cost of $ 1,821 [4]. The discrepancy between hospital and community acupuncture use suggests that acupuncture is under-utilised in hospital settings.

### Acupuncture research

Amongst Australian general practitioners (GPs), acupuncture is one of the most accepted forms of complementary medicine [5]. In 1998, a United States National Institute of Health Consensus Conference Panel reviewed the status of acupuncture and concluded that: *"There is sufficient evidence of acupuncture's value to expand its use into conventional medicine and to encourage further studies of its physiology and clinical value*."[6] Similarly, in 2002, the World Health Organisation (WHO) stated that acupuncture is a safe, simple and convenient therapy and that its effectiveness as analgesia has been established in controlled clinical studies.

While the literature on the physiological effects and mechanisms of acupuncture analgesia is extensive, the clinical literature is of poorer quality [2,6,7]. Most reports have methodological flaws including poor study design, inadequate statistical analysis, difficulties in diagnosis definition, unsuitable placebo or sham interventions and control groups, and a lack of funding, or qualified researchers with access to clinical settings [7,8].

Notwithstanding these difficulties, it has been shown that acupuncture analgesia in the treatment of chronic pain is comparable to morphine and that its better safety profile and lack of dependence makes it the preferred method of choice for these conditions [9].

Also, the publication of the Standards for Reporting Interventions in Controlled Trials of Acupuncture (STRICTA) guidelines in 2002[10] has resulted in a dramatic improvement in the precision of reporting of acupuncture interventions [11].

There are very few clinical trials of acupuncture for acute pain to inform clinical practice [7]. When reviewing the evidence for the use of acupuncture in acute settings, only two published randomized controlled trial of acupuncture in an ED setting could be located in the peer-reviewed literature [12,13]. One study found a significantly greater reduction in pain in the group receiving acupuncture and standard therapy, compared to the group receiving standard therapy alone [12]. The other found that the use of concomitant auricular and electrical acupuncture, when implemented early in acute spinal cord injury, can contribute to significant neurologic and functional recoveries [13]. Although these studies describe the acupuncture treatment protocol and utilised some blinding (blinding of assessors) they did not examine the effect on length of stay (ED and hospital), applicability or acceptability of acupuncture.

As of June 2011, virtually all of the 31 reviews and 32 review protocols listed in the Cochrane library suggest that the evidence for acupuncture is inconclusive. Consequently, there has been a call for the funding of well-planned, large-scale studies to assess the effectiveness and cost-effectiveness of acupuncture under real-life conditions [14]. It has been suggested that the best methodological approach to clinical acupuncture research is through pragmatic trials where acupuncture is compared to standard care rather than placebo-controlled trials. Such trials may be more able to provide data that is relevant to patients, practitioners and policymakers, and to inform decision-making about treatment options [15].

### Aims

The primary aim of this research is to determine whether acupuncture is effective, safe and acceptable for patients presenting to the ED with low back pain, migraine or acute ankle injuries. The impact on health resource utilisation will also be explored.

### Methods

This project comprises a suite of multicentre, pragmatic, single-blinded, randomised, and controlled clinical trials. They will compare the effects of acupuncture alone, pharmacotherapy alone and acupuncture/pharmacotherapy combined for the three conditions of interest (ankle sprain, migraine or lower back pain).

The trial is registered with The Australian New Zealand Clinical Trial registry and received approval from the ethics committees of RMIT University, as well as the participating hospitals.

### Population

Patients will be recruited through the EDs of four large tertiary teaching hospitals in Melbourne, Australia. These include two public EDs (The Northern Hospital and the Alfred Hospital Emergency and Trauma Centre) and two private EDs (The Epworth and Cabrini Hospitals). Participants will be recruited over a period of approximately 24 months with a target sample size of 505 subjects.

### Inclusion Criteria

Patients aged ≥ 18 years and who require analgesia for one of the following conditions will be considered for enrolment:

1) *Low back pain: *all patients with either acute or acute-on-chronic low back pain, with or without referred pain. For the purposes of the study, acute low back pain is defined as pain of less than 3 months duration [16]

2) *Migraine: *all patients with migraine, as defined by the International Headache Society [17].

3) *Acute ankle injuries*: all patients with an acute ankle injury within 72 hours of presentation.

### General Exclusion Criteria

Patients will be excluded from all three studies if any of the following is applicable:

1) temperature > 37.7°C[18]

2) acute major trauma, such as falls from a height or road traffic accidents

3) anticoagulation medication use or the presence of a mechanical heart valve

4) skin infections that would preclude certain acupuncture points being used

5) signs of illness that the treating physician feels will make them inappropriate for acupuncture or pharmacotherapy

6) refusal, inability to consent or communication difficulties

7) any form of analgesia up to 60 minutes prior to t0 (study commencement)

8) an initial pain score ≤ 4 on the pain scale (score range 0-10)

9) presentation to the ED > 4 times in the previous 3 months with the same condition

### Specific Exclusion Criteria

1) *Low back pain trial: *any suspected non-musculoskeletal cause for the back pain and suspected vertebral fracture.

2) *Migraine trial: *any suspected condition not consistent with the definition of migraine

3) *Acute ankle injury trial: *obvious deformity suggestive of ankle dislocation or fracture.

An ED physician will assess each patient and, if they meet the study entrance criteria, informed consent will be obtained. The patient will then be randomised to either acupuncture, pharmacotherapy or acupuncture/pharmacotherapy combined.

### Interventions

#### Acupuncture Alone

The acupuncturists will be either a qualified Traditional Chinese Medicine TCM practitioner registered with the Chinese Medicine Registration Board of Victoria or an ED physician with medical acupuncture qualifications accredited with the Royal Australian College of General Practitioners and the Australian Medical Acupuncture College (RACGP-AMAC) Joint Consultative Committee on Medical Acupuncture.

Treatment protocols were determined through review of major clinical manuals and textbooks, literature review, and a panel of specialist acupuncturists from both western medicine and Chinese medicine backgrounds. The protocols, which allow acupuncture points to be selected from a pool of pre-determined points for each condition, provide sufficient standardisation to assist replication, yet are flexible enough to allow individualised treatments (Table 1). These protocols also allow for additional points, such as 'ashi points', to be used at the discretion of the acupuncturist. The location of the points, angle of insertion and depth of insertion were sourced from a popular text 'A Manual of Acupuncture' [19].

**Table 1:** Acupuncture point selection protocol

	Local points	Distal points	Other	Stimulation	Needle choice and Retention Time
**All three Conditions**	LI4 and LV3				Hwato 0.22 × 13 mm Hwato0.25 × 30 mm Hwato0.25 × 40 mm Seirin 0.25 × 30 mm 20-30 minutes retention

**Lower Back Pain:** **Central type**	Huatoujiaji, DU3 and DU4, BL 29, BL30, BL31 and BL32	GB 30, BL40, BL54, BL 58, BL62, and KI 3	Other points- Ashi points as suitable	- Local and Ashi points should be stimulated gently til Deqi is achieved or in the case of a trigger point where a twitch response is elicited.	
		
**Lower Back Pain:** **Lateral Type**	BL23, BL25, BL52, BL54 and Yaoyan	GB 30, BL40, BL54, BL 58, BL62, and KI 3	Other points- Ashi points as suitable	- Distal points stimulated more strongly and using a reduction technique	
	
**Ankle Sprain**	Local points should be selected from ST41, GB40, BL60, BL 62, BL 63 or 64 KI2, KI3, KI6, SP4, SP5	Distal points SP6, SP9, GB34, ST36 (use with caution on patients with low blood pressure) and HT7 on the opposite wrist to the injury	Other points- Ashi points as suitable	- Local and Ashi points should have no or very little stimulation - Distal points are to be stimulated more strongly using a reduction technique	
	
**Migraine**	-Minimal local point selection (1-2 points) from Taiyang, ST8, GB8, DU23, BL10, BL 11, GB2 and GB14 according to pain location. If significant retro-orbital pain: BL2	-Distal points use LV3, LV 2, GB34, LI4, SJ5, ST 36, ST44, BL60, GB41, SP6	Nausea/vomiting add, GV18, PC6 or ST40 Other points- Ashi points as suitable	-Local points should be no/minimal stimulation -Distal points should use a reduction technique	

#### Pharmacotherapy Alone

Patients in the pharmacotherapy group will receive treatment as per standard practice within each ED and good clinical practice guidelines. These are based on guidelines from the National Institute of Clinical Studies (NICS) and National Health and Medical Research Council (NHMRC) for migraine [20,21] and NHMRC for low back pain and ankle injuries [22].

From an expert panel of emergency physicians, a drug therapy protocol was developed based on the most commonly administered analgesics. While comprising 1st and 2nd line drugs, it was recognized that while patients should be treated with one of the drugs from the protocol, other drugs may be administered at the treating physicians' discretion. This may occur in cases of known drug allergy, previous efficacy of other drugs for the individual or personal preference of the physician (Table 2).

**Table 2:** Pharmacotherapy selection protocol

1st line therapy options:	*Ankle sprain*	*Lower back pain*	*Migraine*
diazepam 5 mg		X	

3centerHartmans5% DextroseNaCl 0.9%	X	X	X

metaclopramide 10-20 mg IV or prochlorperazine 12.5 mg IM, if there is significant nausea and vomiting.			X

paracetamol 1 g	X	X	X

paracetamol with codeine	X	X	X

Tramadol 50-100 mg	X	X	X

dextropropoxphene and paracetamol	X	X	X

ibuprofen OR diclofenac OR indomethicin	X	X	X

**2^nd ^line therapy options:** **> 1 hour (t1)**	***Ankle sprain***	***Lower back pain***	***Migraine***

Morphine 2.5 mg IV boluses	X	X	X

Chlorpromazine: 25 mg in 1000 ml normal saline IV			X

#### Combined treatment

Patients in the combined therapy group will be administered both acupuncture and pharmacotherapy, as described above. In order to maintain acupuncturist blinding, the acupuncture will be either administered before or after the pharmacotherapy rather than concurrently. After one treatment has commenced an allowance of up to 15 minutes will be permitted for the second treatment to follow. Administering the second treatment outside the 15 minutes will be considered a violation of protocol and the data will be excluded.

#### Specific treatments

As the mainstay of immediate treatment for acute ankle injuries is rest, ice, compression and elevation (R.I.C.E.), all ankle sprain patients will receive this management regardless of their randomisation status. Similarly, as vomiting and potential dehydration is a common feature of patients presenting with migraine, migraine patients will receive intravenous fluids at the treating physicians' discretion, regardless of their randomization status. Rescue therapy (pharmacotherapy or acupuncture) will be allowed for patients with inadequate pain relief after 1 hour (t1), or earlier if the treating physician advises.

### Randomisation, Blinding and Comparator

Randomisation will be undertaken by an independent statistician using a computerized software randomization program. Each condition and site will be stratified and block randomised, respectively, with the treatment allocation concealed in sealed opaque envelopes. These envelopes will be stored at each site in a locked filing cabinet, until the researcher has screened the patient for inclusion and gained signed informed consent. At this time, the next envelope in sequence will be accessed and opened by the triage nurse (not the researcher).

As this is a pragmatic trial, participants will not be blinded to their treatment allocation [23]. However, the study will be single-blinded such that the researcher performing outcome measure assessments will be blinded to the patient's treatment allocation. Furthermore, the acupuncturist will be blinded as to whether the patient is also receiving pharmacotherapy.

### Data collection

Data will be collected hourly from baseline (t0, t1, t2, etc) and at discharge unless data was collected within the 20 minutes of discharge. A telephone follow-up will be conducted within 48 ± 12 hours of ED discharge, by a researcher blinded to the participant's treatment allocation. **(**Table 3**)**Participation in the study will end at any stage if the patient refuses to continue or in the presence of significant clinical deterioration, as determined by the attending ED physician. For those participants who are lost to follow up or drop out, after t1 data has been collected, intention to treat analysis will be applied to the existing data.

**Table 3:** Data measures

	t0	t1	t2 >	Discharge	48 ± 12 hours post discharge
**Demographics (age, post code, triage category)**	**X**				

**Triage time, time seen by doctor, time of discharge**				**X**	

**Pain scores (at rest, at movement)**	**X**	**X**	**X**	**X**	**X**

**Signs and symptoms**	**X**	**X**	**X**	**X**	**X**

**Relative instrument (MQOL, Global Ankle Scale or OSWESTRY)**	**X**	**X**		**X**	**X**

**Acceptability, willingness to repeat**		**X**		**X**	**X**

**Complications of acupuncture**		**X**		**X**	**X**

**Additional medication used and health care utilization**					**X**

### Primary Outcome Measures

1) Pain

Pain (at rest) will be assessed using a Verbal Numerical Rating Scale (VNRS) out of 10 (0 = no pain, 10 = worst pain imaginable) [24].

The three treatment groups will be compared as a function of the proportion of patients

who have clinically significant pain score reductions, as follows:

a. to < 4/10 (mild pain)

b. of > 2/10 (a clinically significant pain reduction)

### Secondary Outcome Measures

1) *Functionality*:

a. Back pain: Oswestry low back pain disability questionnaire [25].

b. Migraine: 24-hour Migraine Quality of Life questionnaire [26].

c. Acute ankle injuries: Patient's Global Assessment of Ankle Injury Scale [27].

2) *Adverse events:*

a. Nausea, dizziness, drowsiness, fatigue, rashes, visual disturbance, constipation, abdominal pain, and other adverse events using a rated 0-10 (no impairment - intolerable)

c. Vomiting: documenting the number of times vomited since last assessment

d. local complications of acupuncture; bleeding, bruising or other complication

3) *Acceptability: *will be assessed at t1, on leaving the ED and at 48 hour follow up using the follow instruments:

a. Satisfaction: using an ordinal scale 0-10 (0 = very dissatisfied, 10 = very satisfied)

b. Willingness to repeat similar management in future: using a 5 option ordinal scale (definitely no, probably no, unsure, probably yes, definitely yes).

4) *Health Resource Utilization*: will be determined after the 48 hour follow up using the following variables:

a. Length of stay in the ED

b. Length of stay in the Hospital

c. Admission rate

d. Re-presentation rate

e. Seeking other Health Care Professional advice after initial presentation.

f. Additional analgesia/pharmacotherapy used after initial presentation (prescription as well as non-prescription)

5) *Rescue therapy requirements in the ED:*

a. At t1

b. At any stage following t1

#### Safety and Ethics

A Data Safety and Monitoring Committee (DSMC), independent of the investigators, comprising experts in clinical trials, biostatistics and acupuncture/emergency medicine will be established before patient enrolment. The DSMC will review all trial protocols, monitor patient safety, investigate any adverse events, and analyse mid trial results. They will terminate the trial if there are concerns for participant safety, or if the interim results indicate that a particular arm of the trial is clearly superior to the others.

#### Statistical methods

We performed sample size calculations for each of the three conditions, i.e., back pain, migraine and ankle sprain, using both the equivalence (between acupuncture and pharmacotherapy) and non-inferiority (between combined and pharmacotherapy) approaches, assuming that a difference of 1.5 units in Visual Analogue Scale (VAS) score is clinically relevant. The conservative equivalence sample size will allow detection of superiority of the combined treatment arm if present.

Standard deviations were estimated from pilot data obtained from the Northern Hospital. Table 4 was generated using sample size software (PASS2005; Utah; USA). It shows the number of participants required per intervention group (round to the next 5) to achieve 0.8 power at the 0.05 alpha level, after adjusting for an attrition rate of no more than 10%. Hence, the maximum number of subjects required will not exceed (70 + 60 + 55) × 2 + (35 + 35 +65) = 505. We also conducted a sensitivity analysis and found that the maximum sample size will reduce to 360 if the equivalence limit is defined as 2.0 units of VAS.

**Table 4:** Sample size justification

Testing		Power	Sample Size	Equiv Limit	True Diff	Standard Deviation
**Equivalence**	**Ankle Sprain**	0.81	35	1.5	0.0	1.95

	**Migraine**	0.80	35	1.5	0.0	1.96

	**Low Back Pain**	0.80	65	1.5	0.0	2.79

**Non-Inferiority**	**Ankle Sprain**	0.80	70	1.5	0.0	3.25

	**Migraine**	0.80	60	1.5	0.0	2.98

	**Low Back Pain**	0.81	55	1.5	0.0	2.88

#### Data analysis

Descriptive statistics and 95% confidence intervals will be reported. Continuous outcome variables, of the groups at different time points, will be compared using mixed models with the treatment group as a fixed factor and time as a random factor. Categorical data will be analysed using generalized estimating equations (GEE). While all analyses will be undertaken following the intention-to-treat principle using imputation techniques, per-protocol analysis will also be undertaken as a form of sensitivity analysis. The Sharpened Bonferroni method will be used to adjust for individual alpha level when multiple testing is performed. For qualitative data, we will identify recurring themes and develop a conceptual framework to apply to the complete data set. Data analysis will be conducted by specialist statisticians' independent from the research team.

## Discussion

The results of this research will determine if acupuncture, alone or as an adjunct to pharmacotherapy provides effective, safe and acceptable pain relief for patients presenting to EDs with acute back pain, migraine or ankle sprain. The results will also identify the impact that acupuncture treatment may have upon health resource utilisation in the ED setting.

These results could inform revisions of ED clinical pain management guidelines as well as the training of emergency physicians and ED staffing on a national and international level.

The results could also provide consumers with information about viable analgesic choices. If acupuncture is demonstrated to be effective in these conditions, there may be a number of patients with these conditions who seek out acupuncture rather than pharmacotherapy.

The research may also have a major impact on future research for other acute symptoms where acupuncture is thought to be efficacious.

## Trial status

The trial is currently in recruitment phase.

## Competing interests

The authors declare that they have no competing interests.

## Authors' contributions

MC conceived of the study, prepared the initial protocol and drafted the manuscript. SP drafted the manuscript, made amendments to the protocol and developed the acupuncture point protocol. DT drafted the manuscript, participated in design of the study, and participated in development of the pharmacotherapy selection protocol. DV drafted the manuscript, participated in design of the study, and participated in development of the pharmacotherapy selection protocol. MB drafted the manuscript participated in design of the trial protocol, participated in development of the pharmacotherapy selection protocol and participated in the development of the acupuncture point protocol. PC drafted the manuscript, participated in design of the study, and participated in development of the pharmacotherapy selection protocol. CX drafted the manuscript and participated in design of the study. All authors read and approved the final manuscript.
